# The Surgical Anatomy of the Arteries, and Descriptive Anatomy of the Heart; Together with the Physiology of the Circulation in Man and Inferior Animals

**Published:** 1839-10

**Authors:** 


Art. X.—*The Surgical Anatomy of the Arteries, and Descriptive Anatomy of the Heart; together with the Physiology of the Circulation in Man and Inferior Animals.* By Valentine Flood, a.m., m.d., t.c.d. &c.; and Lecturer on Anatomy and Surgery in the North London School of Medicine.—*London*, 1839. 12mo, pp. 237.

The present work, although consisting of but one volume, and that not a very thick one, is characterized by being more comprehensive in its reach than any of its predecessors on the same subject, in the class of *manuals;* indeed, whatever fault we might be disposed to find with its author, we cannot accuse him of having begun in the middle of his subject. The introduction, which extends through some sixty pages, presents us first with Cuvier's classification of animals; and the circulation in each class is considered, commencing from the zoophytes, and proceeding upwards in the scale till we arrive at mammalia. The circulation in man being thus ushered in, the physiology and development of the propelling and conducting organs of the blood are then discussed. The descriptive anatomy of the heart succeeds, and prepares us for the next and grand division of the work, "the Descriptive Anatomy of the Arteries." Considerable care and attention seem to have been bestowed on the compilation of the different divisions which compose this work, particularly in collecting the best information relating to varieties in the course of important arteries; and the more momentous operations on these vessels are illustrated by a selection of the most remarkable cases: the opinions of able operators have been carefully consulted, and are judiciously commented on. The production is altogether very creditable to its author. We are not, however, disposed to believe that the present volume will supersede the excellent work upon the same subject by Dr. Harrison, whose descriptions are, as we well recollect his vivâ voce teaching to have been, most lucid and satisfactory. Dr. Flood's work, however, comprises much which Dr. Harrison's does not profess to contain; and of necessity, as its bulk is greater, the descriptive department is treated of more at large by the latter author. The distinction of type, adopted by Dr. Flood, for those parts of the text which are necessary whilst dissecting, and such as may be left for after-perusal, will obviate the confusion which might otherwise distract the attention of the student whilst simply tracing the course of an artery.

